# Individual Differences in the Change of Attentional Functions With Brief One-Time Focused Attention and Open Monitoring Meditations

**DOI:** 10.3389/fpsyg.2021.716138

**Published:** 2021-10-29

**Authors:** Masaru Tanaka, Ryoichi Nakashima, Kentaro Hiromitsu, Hiroshi Imamizu

**Affiliations:** ^1^Department of Psychology, Graduate School of Humanities and Sociology, The University of Tokyo, Tokyo, Japan; ^2^Japan Society for the Promotion of Science, Tokyo, Japan; ^3^Department of Intelligence Science and Technology, Graduate School of Informatics, Kyoto University, Kyoto, Japan; ^4^RIKEN CBS-TOYOTA Collaboration Center, RIKEN, Wako, Japan; ^5^Cognitive Mechanisms Laboratories, Advanced Telecommunications Research Institute International (ATR), Kyoto, Japan; ^6^Research into Artifacts, Center for Engineering, The University of Tokyo, Tokyo, Japan

**Keywords:** mindfulness, attention, individual difference, focused attention meditation, open monitoring meditation

## Abstract

Mindfulness meditation is increasingly used for clinical treatment and to improve well-being. One of the most fundamental benefits of mindfulness meditation is now considered as enhanced attentional control. Mindfulness meditation is a complex technique but most of its variants consist of a combination of two types of basic meditation practice: focused attention meditation (FAM) and open monitoring meditation (OMM). Although many studies have examined the effect of relatively long-term meditation on attention, some recent studies have focused on the effect of a brief one-time meditation on cognitive processing, including attentional functions. Furthermore, it is necessary to discuss the relationship between the effect of mindfulness meditation on attentional functions and personality traits (especially traits related to mindfulness). This study investigated whether attentional control is improved by a single 30-min FAM or OMM and whether the degree of improvement in attentional functions – alerting, orienting, and conflict monitoring – induced by the meditation varies according to the participant’s trait scores related to mindfulness measured by the Five Facets Mindfulness Questionnaire. Participants were randomly assigned to one of three groups, i.e., FAM, OMM, and no-meditation (noM) groups, and given an Attentional Network Test before and after each 30-min meditation session. Compared with the noM group, there was no overall improvement in attentional functions with either type of meditation. However, there were associations between the change of the alerting function’s score and the personality traits: in the FAM group, alerting scores were negatively associated with the nonreactivity facet of the FFMQ, and in the OMM group, alerting scores were positively associated with describing facet scores of the FFMQ. The results indicate that the effects of meditation methods on attentional functions could depend on the individual’s traits related to mindfulness and that mindfulness meditation could sometimes appear to have no impact on attentional functions.

## Introduction

Mindfulness meditation, firstly developed by Kabat-Zinn (1990), has been described as a mental training technique that enhances one’s ability to intentionally and non-judgmentally concentrate on the “here and now” (e.g., Ivanovski and Malhi, 2007; Chiesa et al., 2011). According to the discussion by Tang et al. (2015), mindfulness meditation boosts self-regulation by enhancing attention control, improving emotion regulation, and altering self-awareness, leading to alleviation of mental illnesses and improvement of well-being. In particular, enhanced attentional control is considered to be the most fundamental factor of mindfulness meditation (Hölzel et al., 2011).

Mindfulness meditation is complex technique, and there are many types of meditation. Lutz et al. (2008) proposed that the wide range of meditations could be categorized into two basic styles: focused attention meditation (FAM) and open monitoring meditation (OMM). In other words, FAM and OMM are currently combined in mindfulness-based meditation. FAM requires practitioners to focus attention on a single selected target such as breathing. The aim of FAM is to establish a persistent meta-control state with increased top-down selective attention regulation and a narrower attentional focus on the task at hand (Lippelt et al., 2014; Hommel and Colzato, 2017; Immink et al., 2017), which benefits sustaining attention even in the presence of distracters (Hasenkamp et al., 2012; Colzato et al., 2015a). OMM requires practitioners to keep non-reactive moment-to-moment monitoring of one’s experience without a specific focus of attention. The aim of OMM is to establish a flexible meta-control state with weakened top-down selective attention regulation and a broader attentional focus by accepting various experiences (Lippelt et al., 2014; Colzato et al., 2015a; Hommel and Colzato, 2017), which reduces competition between task-relevant and task-irrelevant information (Immink et al., 2017).

Mindfulness meditation training could alter the activation of brain regions related to attentional control. Previous studies reported greater activation of the anterior cingulate cortex (ACC), which is related to executive attentional control (e.g., van Veen and Carter, 2002), during meditation of experienced meditators compared to that of non-meditators (Hölzel et al., 2007) and during a resting state of participants after a 5-day meditation (Tang et al., 2009). Greater activation can also be found in the dorsolateral prefrontal cortex (PFC), which is related to executive processing, after meditation (Allen et al., 2012). Studies on FAM and OMM reported that these two types of meditation establish distinct attention control states (e.g., Manna et al., 2010; Yordanova et al., 2020). For example, Yordanova et al. (2020) found that enhanced beta coherence in electroencephalographic oscillatory spatial synchronization patterns, which is associated with top-down-controlled processing, was lateralized to the right hemisphere in FAM but to the left hemisphere in OMM. In summary, brain regions related to attentional control show functional changes following mindfulness meditation practice. However, it is not clear whether the changes in the brain actually and directly reflect the change of attentional performance itself. Therefore, it is important to examine the change of attentional performance caused by mindfulness meditation.

Attention has various aspects, such as information selection, maintenance of concentration, efforts, and awareness, and thus the influence of mindfulness meditation on attention could be considered in several frameworks (or models) of attention (cf. Lutz et al., 2015; Tang et al., 2015; Isbel and Summers, 2017). The present study focuses on one of the models in which three different neural networks are involved in attention (Posner and Petersen, 1990). According to this model, attentional functions are divided into three components: alerting, orienting, and conflict monitoring, although they can weakly interact each other (Fan et al., 2009). Alerting is defined as activating and maintaining an alert state for an ongoing task. This function relates to wakefulness and arousal, and the ability to increase response readiness to a target after an external warning stimulus. Alerting is associated with the activation of thalamic, frontal, and parietal regions, which can be related to the brain’s norepinephrine system (e.g., Marrocco et al., 1994; Coull et al., 2000, 2001). Orienting is defined as selecting specific information from various sensory inputs. There are two manners of orienting; exogenous (e.g., an external event captures attention) and endogenous (e.g., a person moves attention toward a specific stimulus). Orienting is associated with the activation of the superior and inferior parietal regions, and subcortical areas such as superior colliculus and thalamus (e.g., Corbetta et al., 2000; Corbetta and Shulman, 2002). Conflict monitoring is defined as resolving conflicting information and/or responses. This function is related to many actions in daily life such as planning, decision-making, and overcoming habitual actions. Conflict monitoring is associated with the activation of the ACC and lateral PFC (e.g., MacDonald et al., 2000; Matsumoto and Tanaka, 2004). The Attentional Network Test (ANT) was developed to measure these three independent components simultaneously and quickly (Fan et al., 2002).

Mindfulness meditation can influence several components of attention. Recent studies on the relationship between mindfulness meditation and cognition (attentional processing, especially components of attention) have reported different results (for review, Lao et al., 2016; Cásedas et al., 2020; Prakash et al., 2020). Among the studies examining the effect of mindfulness mediation on attentional processing using ANT, each study suggested that mindfulness meditation improves each component of attention, i.e., alerting (Oken et al., 2010), orienting (Jha et al., 2007), and conflict monitoring (Tang et al., 2007; Ainsworth et al., 2013). Chiesa et al. (2011) indicated that relatively short-term mindfulness meditation (i.e., a few days or a few weeks) might be related to improvements in conflict monitoring and orienting, whereas long-term meditation (i.e., a few months or years) might be related to improvement in alerting. Different types of meditation training were adopted in the studies described above, so it is important to compare the effects of different types of meditation on attention in order to more specifically investigate the effects of meditation. In particular, it is essential to examine the effects of the basic meditation methods (i.e., FAM and OMM) in detail, since many mindfulness meditation programs are composed of a combination of them (Lutz et al., 2008).

Many studies on the effects of mindfulness meditation on cognitive processing involved complicated, long-term training (e.g., multiple-session training and full immersion). However, it is also important to examine the impact of a brief meditation (in other words, a single session meditation) for the following reasons. First, many meditation programs involve repetition of a brief one-time meditation over days, weeks, or months. That is, a brief one-time meditation should be a minimum unit of meditation. Second, it is important to investigate the effect of easy short-duration meditation given the use of meditation in fields such as education and sports (e.g., to improve concentration).

Recent studies have investigated the effect of a brief one-time meditation, such as a brief FAM/OMM on cognitive processing (e.g., Colzato et al., 2015b, 2016; Chan et al., 2017; Baranski, 2021), and indicated that single-session FAM and OMM could have different kinds of impact on cognitive processing. For example, Chan et al. (2017) reported that a single-session FAM (22-min) leads to higher performance of cognitive control in a motor sequential learning task. In addition, Colzato et al. (2016) suggested that a single-session FAM (17-min) modulates the ability to suppress task-irrelevant information by using a global–local task that measures the processing of global/local characteristics of hierarchically constructed visual stimuli (e.g., Navon, 1977). Related to attentional processing, Colzato et al. (2015b) reported that attentional blink, when two target stimuli appear temporally closely in a rapid stream of events and the second target stimulus is often unnoticed, was attenuated after a brief OMM (17-min), indicating that the OMM modulates attentional allocation over time. It should be noted that, however, subsequent study failed to replicate the advantage of the OMM (Sharpe et al., 2021). Moreover, Norris et al. (2018), focusing on conflict monitoring, indicated that 10-min classic mindfulness meditation could improve attentional allocation in novices. Thus, even a brief one-time meditation (FAM and OMM) could influence attentional processing (i.e., the components of attention: alerting, orienting, and conflict monitoring), although not so many studies (compared to the long-term meditation studies) have examined the effect of a brief meditation and little has been clarified about this issue.

It is also important to consider individual differences, that is, the meditation effect may differ based on personality traits and so on (Lippelt et al., 2014; Tang et al., 2015). Recent studies have indicated that the attentional processing performance itself can depend on dispositional mindfulness (Di Francesco et al., 2017; Sørensen et al., 2018), which is named as “personality traits related to mindfulness” in this study. In addition, Norris et al. (2018) indicated that the effect of a brief mindfulness meditation on attention could vary based on personality traits (in that study, neuroticism). Taken together, it is possible that change in the components of attention by mindfulness meditation also depend on personality traits related to mindfulness.

In sum, the aims of the present study are as follows. First, we investigated whether attention would be improved by a single 30-min FAM or OMM session (i.e., a brief one-time meditation or a single-session meditation). The instructions on meditation were prepared by Fujino et al. (2019). They made these instructions, which can be used with naïve participants, based on the identification of meditation techniques and functions of FAM and OMM. In addition, their instructions were made based on the auditory instructions of Colzato et al. (2012), which were used in Colzato et al. (2015a,b) to examine the effect of meditation on attentional control. Furthermore, Ooishi et al. (2021) confirmed that these instructions should be appropriate to change the participants’ states. These studies indicated that the instructions on meditation (Fujino et al., 2019) can establish FAM and OMM states in novices with no prior experience of mindfulness meditation. Although beginners of meditation often feel difficulty in practicing OMM (Malinowski, 2013; Tsai and Chou, 2016), appropriate instructions could help participants to experience mindfulness states even with a one-time practice session. Actually, Colzato et al. (2015b) reported that a single-session OMM influenced the performance of an attention task, although this may not be a robust result (Sharpe et al., 2021). Our interest in this study was the *change* of attentional processing by mindfulness meditations. For this purpose, we conducted ANTs before and after 30-min meditation and calculated the change of performance in three meditation groups, i.e., FAM, OMM, and no-meditation (noM).

We subsequently investigated the relationship between change of attentional functions (i.e., the change in ANT scores for each participant) and participants’ properties related to mindfulness determined by the Five Facet Mindfulness Questionnaire (FFMQ). FFMQ includes five components related to mindfulness (Sugiura et al., 2012; see also Baer et al., 2006): Observing (observing or noticing sensations, perceptions, thoughts, and feelings); Nonjudging (not judging one’s experience); Describing (the tendency to describe or label everything with words); Nonreactivity (nonreactivity to inner experience); and Awareness (the tendency to act with awareness). In this study, we only hypothesized that participants’ properties related to mindfulness (measured by subscales of FFMQ) could influence the changes in attentional functions. We did not propose any specific prediction of results, e.g., a specific subscale being related to a specific component of attention improvement. Rather, we aimed to examine the relationship between traits related to mindfulness and attentional improvement exploratorily.

## Materials and Methods

### Participants

Ninety-six undergraduate and graduate students at the University of Tokyo (64 males; 19–26 years old) participated in this study. All participants reported normal or corrected-to-normal vision and they had no prior formal experience with meditation practice before this experiment. They were randomly assigned to one of three groups (*n*=32 in each group): FAM group (27 males; 20–22years old), OMM group (17 males; 19–26years old), and noM group (20 males; 19–23years old).

Although we did not conduct a power analysis before this study, we reviewed the sample sizes of previous studies examining the effect of 8-day (relatively short-term) FAM/OMM on attention using ANT (Ainsworth et al., 2013: *n*=24) and the effect of 10-min (brief one-time) meditation on attention (Norris et al., 2018: *n*=29). We assumed that the number of participants in each group (*n*=32) is sufficient. It should be noted that this study is a part of the larger study investigating the individual differences of attentional functions.

This study was approved by the Institutional Review Board of the University of Tokyo. Written informed consent was obtained from each participant prior to the experiment.

### Questionnaire

We used the Japanese version of the FFMQ (Sugiura et al., 2012). Sugiura et al. (2012) confirmed that this questionnaire is comparable to the original FFMQ questionnaire (Baer et al., 2006), based on the factor analysis and correlation analysis with related measures. Therefore, we regarded this questionnaire as valid for measuring dispositional mindfulness (i.e., personal properties related to mindfulness). The FFMQ was designed to measure the five main subscales of the mindfulness trait as follows.

- “Nonreactivity” represents nonreactivity to inner experience. An example from the questionnaire is, “I perceive my feelings and emotions without having to react to them.”
- “Observing” is the tendency to observe or notice sensations, perceptions, thoughts, and feelings. For example, “When I’m walking, I deliberately notice the sensations of my body moving.”
- “Awareness” is the tendency to act with awareness. For example, “I find it difficult to stay focused on what’s happening in the present.” (a reverse-scored item)
- “Nonjudging” is not judging one’s experience. For example, “I criticise myself for having irrational or inappropriate emotions.” (a reverse-scored item)
- “Describing” is the tendency to describe or label everything with words. For example, “I’m good at finding the words to describe my feelings.”

### Attentional Network Tests

ANTs were conducted in a dark room. Participants were tested individually. They were seated in front of a display, with the head fixed by a chin rest (viewing distance, 57cm). Stimulus presentation and data collection were performed on a Windows PC running MATLAB (Mathworks) with Psychophysics Toolbox (Brainard, 1997; Pelli, 1997; Kleiner et al., 2007). Visual stimuli were presented on a 24-inch LED display (1,680×1,050 pixels, 60Hz; P2217, Dell). Participants responded using a standard 10-key pad.

**Figure 1:**
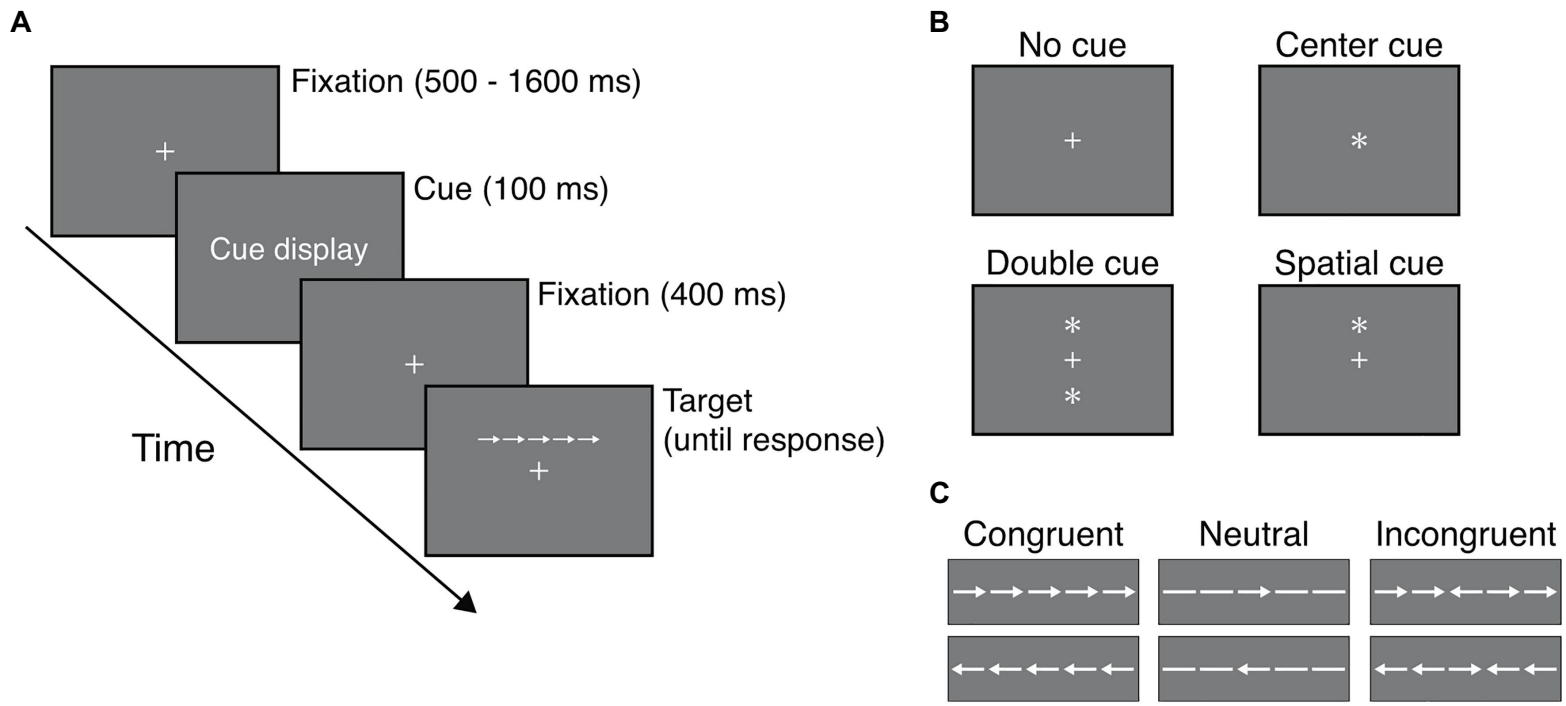
Example of a trial in the Attention Network Test. **(A)** Sequence of trial events. In the “Cue display,” one of the four cues was presented. For the “Target” period, one of the stimuli was presented above or below the fixation cross. **(B)** The four cue conditions. **(C)** The three stimulus conditions. The center arrow of each stimulus was the target.

The ANT comprised cues and a flanker task (Figure 1A). Cues were indicated by small asterisks on the display (Figure 1B). There were four cue display conditions: no cue, center cue, double cue, and spatial cue. Participants saw only the fixation cross (0.55°×0.55°) in the cue display (i.e., the same as the fixation display) under the no-cue condition. The fixation cross changed to an asterisk (0.55°×0.55°) in the center-cue condition. Two asterisks were presented at the two locations corresponding to the two possible target positions (above and below the fixation cross) under the double-cue condition. An asterisk was presented above or below the fixation cross under the center-cue condition. The target always appeared at the location of the asterisk in the spatial-cue condition, and the target positions varied (above or below) randomly in other conditions.

We used the arrow version of the flanker task. There were three types of stimulus conditions in the flanker task: neutral, congruent, and incongruent conditions (Figure 1C). The target was the arrow at the center of the stimulus array. The target was flanked by two lines on both sides in the neutral condition or by two arrows in the other two conditions. The arrows pointed toward the same direction as the target arrow in the congruent condition or in the opposing direction in the incongruent condition. The breadth of the arrow and the line was 0.55° of visual angle. Adjacent arrows or lines were separated by 0.06°. The distance between the center of the fixation cross and the center of the target was 1.5°.

Figure 1A shows the sequence of a trial. Trials started with a white fixation cross located at the center of a uniform grey display for random variable duration (500–1,600ms), and participants were told to gaze at the center of the display. The fixation cross was followed by a cue display lasting 100ms, and then the fixation cross was presented again for 400ms. Next, stimuli including a target appeared above or below the fixation cross. Participants were asked to report whether the target arrow pointed to the left or the right as fast and accurately as possible by pressing “4” for left and “+” for right on a standard 10-key pad. The 10-key pad was placed in front of the participants, who pressed the “4” and “+” keys using their left and right index fingers, respectively. The inter-trial interval was 1,000ms.

Each ANT included 288 trials, lasting about 20min. Participants were allowed to rest freely every 48 trials, which consisted of three stimulus types×two target locations×two target directions×four cues. The trial order was randomized across participants. A practice block (24 trials) was conducted prior to the pre-meditation ANT to allow participants to become familiar with the task. We planned for participants initially showing low performance to repeat the practice block until the accuracy rate exceeded 80%, but no participants required an additional practice block. In the practice blocks, a beep sound was audible in the headphones (ATH-AR3, Audio-Technica) when participants responded incorrectly.

### Meditation Sessions

All meditation sessions lasted 30min. During the session, all participants (including noM group) sat on a chair and listened corresponding sounds (i.e., meditation instruction or music) on headphones, with their eyes closed. Participants in the FAM and OMM groups practiced the corresponding meditation, listening to Japanese auditory instructions spoken by a highly experienced meditation instructor, and following the instruction. The detailed instructions were obtained from Fujino et al. (2019). In the FAM group, participants practiced concentration by staying focused on their own breathing. In the OMM group, participants practiced awareness of their experiences through simply observing and feeling their sensations, emotions, and thoughts without judgment or reaction. The participants in the noM group just listened to the music, which is a commercially available CD of relaxation music including natural sound and BGM (EAN: 4961501643379). Participants in the noM group did not receive any information about meditation.

### Procedure

First, the paper-and-pencil version of the FFMQ (Sugiura et al., 2012) was conducted. Then, after a 1-min rest, participants underwent a pre-meditation ANT. Next, they practiced the meditation corresponding to their group allocation. Finally, after a 1-min rest from the meditation practice, they underwent a post-meditation ANT. The pre-meditation ANT and post-meditation ANT were completely the same task.

### Data Analysis

We planned to exclude the data of participants whose mean accuracy in the pre-meditation ANT was below 80%, but all participants surpassed 80%. We also planned to exclude trials from the analysis as outliers if any of the reaction times (RTs) in each trial was more than three standard deviations from the individual mean for each cue and stimulus type condition. However, this did not occur and thus no trials were excluded. RTs in the trials with correct responses were analyzed. In addition, we discarded the data of participants whose scores were more than three standard deviations from the group mean in the analyses. All data analyses were conducted using R Ver. 3.6.0 (R Core Team, 2016). The significance threshold was set to *p*<0.05 for all analyses.

Three attention scores are defined by differences in RTs between a set of conditions (cf. Fan et al., 2002). By calculating the difference in RTs, it is possible to eliminate the influence of motor responses from the attention scores. The alerting score was calculated by subtracting the mean RT under the double cue condition from that under the no cue condition. The orienting score was calculated by subtracting the mean RT under the spatial cue condition from that under the center cue condition. For alerting and orienting, larger positive scores indicate more efficient functions because these scores show the degrees of facilitation by the cue. The conflict monitoring score was calculated by subtracting the mean RT under the congruent condition from that under the incongruent condition. For conflict monitoring, larger positive scores indicate that observers take a longer time to resolve conflicts, i.e., poorer function. We calculated these scores and compared the scores between the pre-meditation and post-meditation ANTs. Alerting, orienting, and conflict monitoring scores were individually analyzed using mixed-design analysis of variance (ANOVA) with meditation group (FAM, OMM, and noM) as a between-subject factor and time (pre- and post-meditation ANT) as a within-subject factor. When a significant effect was noted, we planned to conduct multiple subsequent comparisons using Shaffer’s modified version of the sequentially rejective Bonferroni procedure. However, there were no significant effects, and we did not conduct multiple comparisons.

In addition, we investigated whether the five factors in the FFMQ could be related to the change (difference) in each attentional function between the pre- and post-meditation ANTs for each participant (i.e., the post-meditation score minus the pre-meditation score in each function). Specifically, we conducted stepwise linear regressions because the five factors in the FFMQ tend to be highly correlated with each other, and multicollinearity should thus be avoided.

## Results

### Group-Level Comparisons

**Figure 2:**
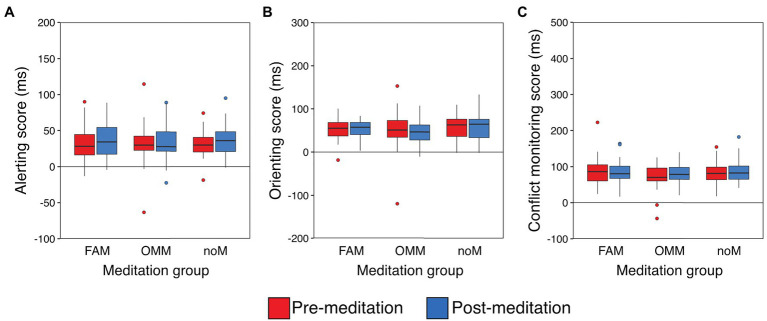
Attentional function scores in pre- and post-meditation ANTs for the three meditation groups after excluding outliers (the scores were more than three standard deviations from the group mean), FAM: focused attention meditation, OMM: open monitoring meditation, noM: no meditation. **(A)** Alerting scores (FAM: *n*=32, OMM: *n*=30, noM: *n*=32). **(B)** Orienting scores (FAM: *n*=32, OMM: *n*=31, noM: *n*=31). **(C)** Conflict monitoring scores (FAM: *n*=31, OMM: *n*=31, noM: *n*=31). Red and blue boxes and circles show the results of the pre- and post-meditation ANT, respectively. In each box-and-whisker plot, the central horizontal line indicates the median, and the bottom and top edges indicate the first and third quartiles, respectively. The upper whisker extends from the edge to the largest value within 1.5 times the interquartile range (IQR) from the edge. The lower whisker extends from the edge to the smallest value within 1.5 times the IQR of the edge. Data points beyond the ends of the whiskers are plotted individually.

All the participants completed the ANT practice in a single block. In the pre- and post-meditation ANTs, accuracies were very high (the mean accuracy under each condition exceeded 90.6%; see Appendix A for details). Thus, we can discuss our findings based on the RT results. Performance, as indicated by the alerting, orienting, and conflict monitoring scores, is summarized in Figure 2. It is noted that there were data of some participants removed from the analyses due to the criterion described above. In alerting, data of two participants were removed from the OMM group. In orienting, data of two participants were removed, one from the OMM group and one from the noM group. In conflict monitoring, data of three participants were removed, one from each group.

We conducted mixed-design ANOVA with time and meditation group factors. We used the Greenhouse–Geisser correction for violations of the sphericity assumption (Geisser and Greenhouse, 1958). In these analyses, our main interest was to examine whether the effect of meditation on attention differed according to meditation type (i.e., the interaction between time and meditation group). For alerting, there were no significant main effects of time, *F*_(1,91)_=3.39, *p*=0.069, *η_p_*^2^=0.037, or meditation group, *F*_(2,91)_=0.01, *p*=0.987, *η_p_*^2^<0.001, and no interaction, *F*_(2,91)_=0.17, *p*=0.843, *η_p_*^2^=0.004. For orienting, there were no significant main effects of time, *F*_(1,91)_=0.004, *p*=0.948, *η_p_*^2^<0.001, or meditation group, *F*_(2,91)_=0.77, *p*=0.468, *η_p_*^2^=0.017, and no interaction, *F*_(2,91)_=0.17, *p*=0.841, *η_p_*^2^=0.004. For conflict monitoring, there were no significant main effects of time, *F*_(1,90)_=1.83, *p*=0.180, *η_p_*^2^=0.020 or meditation group, *F*_(2,90)_=1.00, *p*=0.372, *η_p_*^2^=0.022, and no interaction, *F*_(2,90)_=1.06, *p*=0.352, *η_p_*^2^=0.023. These results showed that a single 30-min meditation did not significantly alter any attentional functions at the group level. No main effect of meditation group and interaction in all analyses indicated that the basic task performance among the three meditation groups were not originally different. It is noted that even if we used data of all participants (i.e., without discarding data of some participants), ANOVAs revealed very similar results (see Appendix B for details).

We conducted *post-hoc* tests for the sample sizes (*n*=32 in each group) with effect sizes of the interaction among the attentional functions using the G*Power software ver. 3.1.9.7 with “Repeated measures, within-between interaction” (Faul et al., 2007, 2009). These tests reveal that the power (1−*β*)=0.178 in both alerting and orienting, and (1−*β*)=0.758 in conflict monitoring.

### Associations Between ANT Changes and Personal Traits Related to Mindfulness

Table 1 shows that mean values of five factors in the FFMQ in each group. The values were not significantly different among groups, *F*s<2.92, *p*s>0.058.

**Table 1:** Means and standard deviations (in parenthesis) of the scores of the facets in FFMQ across groups.

	*FAM*		*OMM*		*noM*	
Observing	22.1	(5.7)	21.9	(6.7)	22.6	(5.3)
Nonreact	18.7	(4.6)	20.2	(3.9)	17.7	(4.1)
Nonjudging	23.3	(8.0)	25.7	(7.5)	24.6	(6.9)
Describing	24.9	(7.2)	22.6	(8.4)	20.7	(4.8)
Awareness	24.8	(6.4)	24.5	(6.8)	24.8	(5.8)

To investigate whether traits related to mindfulness are associated to changes in attentional performance from the pre- to post-meditation ANT, we conducted stepwise linear regressions for each attentional function in each meditation group (see Table 2). For stepwise linear regressions, the independent variables were the five factors in the FFMQ, while the dependent variable was the difference in each attentional function score between before and after the meditation (i.e., the post-meditation score minus the pre-meditation score in each function). Correlation coefficients among the factors in the FFMQ are shown in the Appendix C.

**Table 2:** Results of stepwise linear regressions in the combinations in which changes in ANT score could be predicted by FFMQ factors (after removing outliers).

Independent variable	*r*	*β*	*R^2^*	*adjR^2^*	*F*	*p*
(A) Alerting in the FAM group (*n*=32)						
Nonreactivity	−0.38	−0.38	0.15	0.12	5.18	0.030
Nonjudging	−0.33					
Observing	0.19					
Awareness	−0.17					
Describing	−0.02					
(B) Alerting in the OMM group (*n*=30)						
Describing	0.40	0.40	0.16	0.13	5.23	0.030
Nonjudging	0.19					
Nonreactivity	0.18					
Observing	0.13					
Awareness	0.08					

In the header column, factors in the FFMQ are arranged in order of the absolute correlation coefficient. The right columns show correlation coefficients (*r*) between changes in the ANT score and factors in the FFMQ, standardized regression coefficients (*β*), coefficient of determinations (*R*^2^), adjusted coefficient of determinations (adj*R*^2^), *F*-values and values of *p*. (A) In the FAM group, the change in alerting was predicted by nonreactivity. (B) In the OMM group, the change in alerting was predicted by describing.

**Figure 3:**
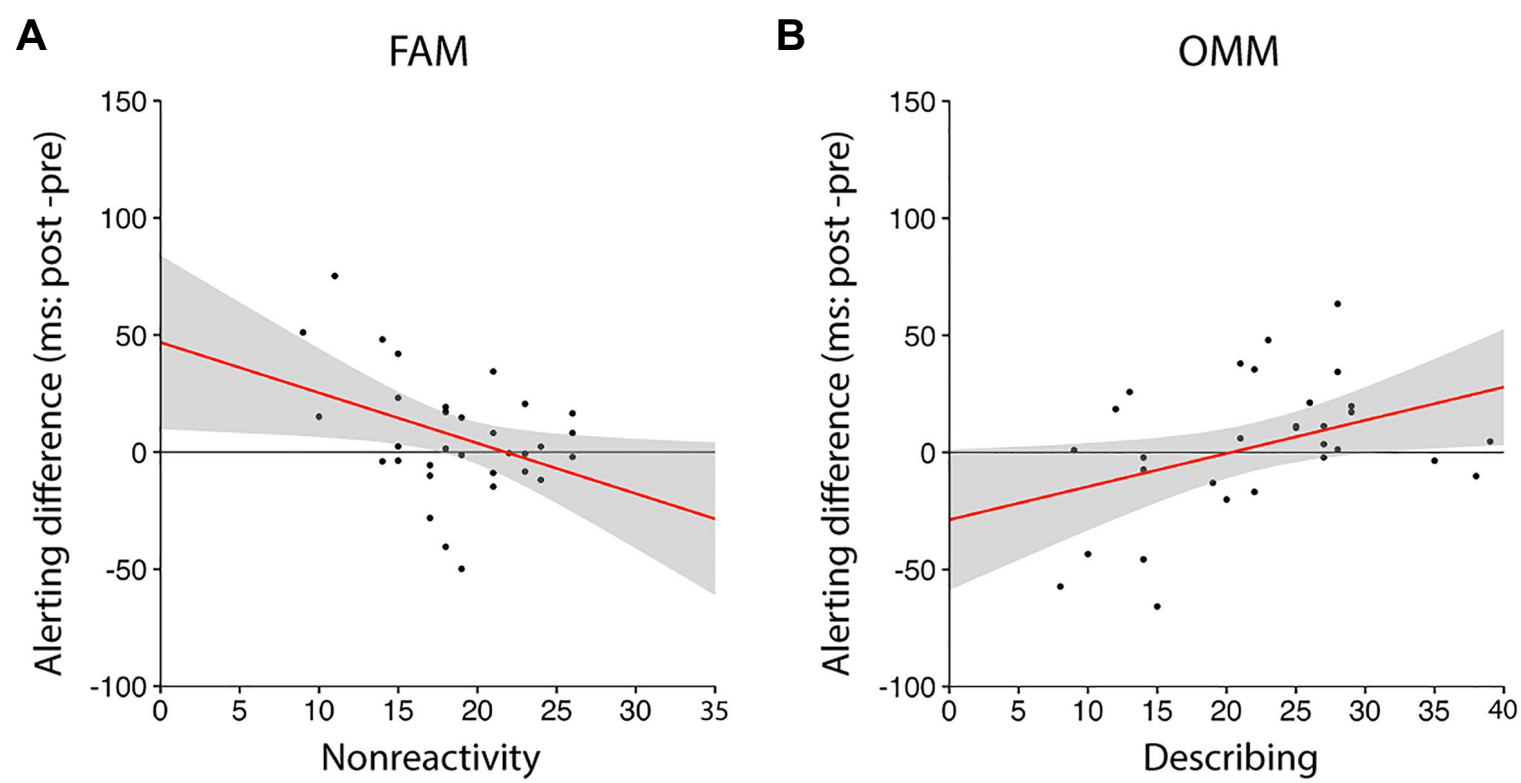
Associations between changes in ANT score and factors in the FFMQ. **(A)** In the FAM group, the change in the alerting score from pre- to post-meditation was significantly correlated with the nonreactivity score (*r*=−0.38, *p*=0.030, see also Table 2). **(B)** In the OMM group, the change in the alerting score from pre- to post-meditation was significantly correlated with the describing score (*r*=0.40, *p*=0.030). Black dots and red lines indicate data of individual participants and linear regressed lines, respectively. Grey shaded areas indicate the 95% confidence intervals of the linear regressed lines.

For alerting in the FAM group, only *nonreactivity* was finally adopted as a significant independent variable (Figure 3A; alerting difference (ms)=−2.15×nonreactivity +46.82, *R*^2^=0.15, *r*=−0.38, *p*=0.030). The negative coefficient indicated that higher alerting change scores in FAM group were associated with lower *nonreactivity* trait scores. For orienting and conflict monitoring in the FAM group, no factor in the FFMQ was adopted as a significant independent variable. For alerting in the OMM group, only *describing* was finally adopted as a significant independent variable (Figure 3B; alerting difference (ms)=1.42×describing −28.77, *R*^2^=0.16, *r*=0.40, *p*=0.03). The positive coefficient indicated that higher alerting change scores in OMM group were associated with higher *describing* trait scores. In the noM group, no factor in the FFMQ was adopted as a significant independent variable. Therefore, we suggest that these facets interact with the specific types of mindfulness meditation.

For orienting and conflict monitoring in the OMM group, no factor in the FFMQ was adopted as a significant independent variable. Here, we obtained similar results when we conducted the analyses using the data of all participants in each group (see Appendix D).

## Discussion

In this study, we investigated the effects of single 30-min (i.e., brief one-time) FAM and OMM on attentional functions. Group-level analyses revealed no improvement in attentional functions in the FAM or OMM group compared with the noM group. We next investigated the relationship between participants’ traits related to mindfulness and the potential change of attentional functions in each meditation group. The regression analyses suggest that the degree of the change in alerting in FAM group is negatively associated with higher scores for nonreactivity, and the change in alerting in OMM group is positively associated with higher scores for describing. These are tentative findings suggesting that change alerting function depends on specific traits related to mindfulness combined with a specific type of mindfulness meditation.

The results of group-level analyses show that no meditation in this study changed any ANT score in general, indicating that a single 30-min meditation session (FAM or OMM) has little/no impact on the change of attentional functions. Previous experiments conducting a single-session meditation session found that the mediation had an impact on cognitive task performance (e.g., Colzato et al., 2015b, 2016). However, they conducted the cognitive task only after meditation, that is, they focused on the cognitive performance itself in the meditation group. In contrast, this study focuses on the change of attentional functions between before and after a single-session meditation. In addition, cognitive functions measured by the tasks are different among studies. Even in Colzato et al. (2015b), examining the effect of meditation on attentional blink, the task was very different from the task in this study. These differences may produce the different results for the effect of a single-session meditation. Furthermore, Sharpe et al. (2021) failed to replicate the results that a brief meditation, especially OMM, influences temporal attention (i.e., attentional blink task), and suggested the greater increase of arousal by the meditation than by the simple relaxation. In summary, there are few robust findings about the effect of a brief meditation on attention, and this study also suggests little/no impact, rather than significant impact, of a brief meditation on attention at the group level. Of course, a single-session meditation could influence especially other cognitive processing (e.g., arousal, global–local processing, etc.) than attentional functions. It is not clear the effect of a single-session meditation yet, and it is important to examine what kinds of processing a brief meditation would influence in order to understand the effects of meditation fully.

Although we did not find any effects of a brief one-time meditation at the group level, we found interesting associations between the participants’ traits related to mindfulness and changes in ANT scores in each meditation group. The results in the FAM group show that the change of the alerting score (a particular aspect of attentional function) tended to be higher as the nonreactivity trait score decreased. The results imply that FAM would change the alerting of participants with lower nonreactivity scores, but not change the alerting of participants with higher nonreactivity scores (Figure 3A). That is, FAM would not influence alerting in persons with a somewhat mindful state, e.g., doing behaviors related to mindfulness daily. The results in the OMM group show that the change of the alerting score tended to be higher as the *describing* trait score increased. The results imply that OMM would change the alerting of participants with higher describing scores, whereas have very little effects on the alerting of participants with lower describing scores (Figure 3B). Especially for alerting, each type of mindfulness meditation (FAM or OMM) has a different impact on the attentional function based on the participants’ personality traits, and thus a group level effect would not appear.

Overall, these results suggest that the change of attentional functions by meditation trainings could depend on the person’s traits related to mindfulness, in addition to the previous suggestion that attentional performance itself could vary depending on the person’s traits (Di Francesco et al., 2017; Sørensen et al., 2018). Assuming that there is interaction between the types of mindfulness meditation training and a person’s traits for improving attentional functions, it seems reasonable that studies examining the effect of meditation on attention at the group level have shown different results (Lao et al., 2016; Cásedas et al., 2020; Prakash et al., 2020). In normal experimental settings, participants are randomly assigned to meditation groups (e.g., FAM, OMM, and noM). That is, in each group, there are many types of participants, from those with lower traits related to mindfulness to those with higher traits. Thus, the results about the effect of meditation can be varied based on the participants’ traits in each group.

Based on the present results and an interpretation of them, we discuss the issues of the effects of short-term and long-term meditation on attentional function. Alerting may change after long-term meditation, such as one-month meditation training (Jha et al., 2007), or may not change after a short-term (about 1-week) meditation (Tang et al., 2007; Kwak et al., 2020). Considering that many mindfulness meditation practices are a combination of FAM and OMM, the long-term meditation may establish the state in which a relatively mindful person practices OMM. Our results that OMM changes the alerting of participants with higher describing scores is similar to those in this situation, and in no way conflicts with previous studies. It is noted that, of course, our results that a brief one-time meditation (FAM and OMM) did not change alerting at the group level is consistent with the suggestion in previous studies that a short-term mindfulness meditation has no/little effect on alerting.

Our results show that a brief one-time meditation has no impact on any attentional component assessed by the ANT, and especially the changes in the orienting and conflict monitoring scores are not associated with any personality traits related to mindfulness. Previous studies reported that several days of mindfulness meditation improve conflict monitoring in particular (Tang et al., 2007; Ainsworth et al., 2013; but see Tsai and Chou, 2016). Therefore, by repeating the mindfulness meditation, we may obtain significant improvement in attentional functions and clear differences between FAM and OMM at the group level. In any case, for practical issues, such as the application of mindfulness meditation to problems in attentional function, it would be important and interesting to determine whether the effect of meditation depends on individual differences in personal traits.

Recently, mindfulness meditation has been introduced into education sites, and its effectiveness has been investigated (Rempel, 2012). Long-term mindfulness meditation interventions were found to improve the attentional functions of students in elementary schools (Napoli et al., 2005) and in colleges (Morrison et al., 2014). These studies indicated that, to improve attentional functions, it is important to maintain the students’ motivation to practice meditation as part of continuing interventions. For students themselves to stay motivated and repeatedly practice meditation, it is desirable for them to appreciate the effect early in meditation. A good way for students to continue meditation without stress is to select the best meditation method based on individual personalities and to help participants to recognize the effect early in the meditation session. Actually, a recent study investigated the relationship between individuals’ personal traits and their preferences for mindfulness meditation (Tang and Braver, 2020). This approach could be applied to sport fields, where mindfulness meditations have been used to obtain various benefits, particularly in terms of concentration (Birrer et al., 2012; Gardner and Moore, 2012).

It is also important to develop more effective meditation methods. Generally, in many mindfulness training programs, beginners practice only FAM, and those who have trained for a long time can practice OMM (Malinowski, 2013; Tsai and Chou, 2016). However, little evidence has been presented for the effectiveness of this sequence, i.e., from FAM to OMM (Lippelt et al., 2014). Our results indicate that, in order to improve attentional functions, FAM is effective for less mindful people, whereas OMM is effective for more mindful people, which supports the effectiveness of the sequence of meditation in mindfulness meditation programs. It should be noted that our observations are based on an exploratory analysis, and these are tentative interpretations. It is necessary to examine in detail the relationship between the effect of mindfulness training and persons’ traits related to mindfulness.

There are some limitations to this study. First, it should be noted that although we did not find any statistically significant change of attentional functions by a brief one-time (i.e., a single-session) mindfulness meditation, these results did not firmly conclude that brief meditations do not have any impact on attentional function. The results of *post-hoc* tests for the sample sizes indicate that the sample size in this study may be too small to detect the effects of a brief one-time meditation on attentional functions at the group level. The effects of a one-time meditation may be very small, even if they exist. Thus, we only tentatively assume that a single-session meditation should have no impact on attention. Further research examining the effect of a brief one-time meditation on attention with larger sample size is necessary, such as Sharpe et al. (2021). Second, it is important to confirm whether participants can establish a mindful state through following instructions on a brief meditation. Although Fujino et al. (2019) aimed to make instructions that could be used with naïve participants (see also Ooishi et al., 2021), we cannot determine whether this succeeded completely. Whether participants could follow the instructions completely and change their state may be one of the limitations of the studies (including this study) examining the effect of a brief one-time meditation. Third, it is important to consider the experimental design, where ANTs were conducted immediately before and after the brief meditation. We believe that conducting the pre- and post-meditation ANTs is essential to clarify the effect of meditation on attentional functions. However, the interval durations (1min in this study) between pre-meditation ANT and meditation and/or between meditation and post-meditation ANT may be too short for participants to change their mind (e.g., from performing a cognitive task to practicing meditation). It may be necessary to make the interval between the ANT and meditation longer in this type of experimental design.

## Conclusion

In this study, we suggest that brief one-time meditations, either FAM or OMM, do not universally improve attentional function. If there is an effect of mindfulness meditation exercises on attentional functions, it is likely to be small and perhaps limited to specific individuals depending on their personality traits related to mindfulness. By considering individual personality traits, it would be possible to investigate the effect of meditation on attention further.

## Data Availability Statement

The original contributions presented in the study are included in the article/Supplementary Material, further inquiries can be directed to the corresponding author.

## Ethics Statement

The studies involving human participants were reviewed and approved by the Institutional Review Board of the University of Tokyo. The patients/participants provided their written informed consent to participate in this study.

## Author Contributions

MT and RN designed the experiments, analyzed the data, and wrote the manuscript. MT, RN, and KH performed the experiments. MT, RN, KH, and HI discussed the results and reviewed the manuscript. All authors contributed to the article and approved the submitted version.

## Funding

This work was supported by KAKENHI grants from the Japan Society for the Promotion of Science (18H05302 to HI and 19K03380 to RN). HI was supported by a KAKENHI grant (19H05725) and a grant for “Research and development of technology for enhancing functional recovery of elderly and disabled people based on noninvasive brain imaging and robotic assistive devices” program for Commissioned Research of the National Institute of Information and Communications Technology (NICT).

## Conflict of Interest

The authors declare that the research was conducted in the absence of any commercial or financial relationships that could be construed as a potential conflict of interest.

## Publisher’s Note

All claims expressed in this article are solely those of the authors and do not necessarily represent those of their affiliated organizations, or those of the publisher, the editors and the reviewers. Any product that may be evaluated in this article, or claim that may be made by its manufacturer, is not guaranteed or endorsed by the publisher.
