# Supplementary material for: Individual Differences in the Change of Attentional Functions With Brief One-Time Focused Attention and Open Monitoring Meditations
Source: Front Psychol. 2021 Oct 29;12:716138. doi: 10.3389/fpsyg.2021.716138 (PMC8585987; doi:10.3389/fpsyg.2021.716138)
Supplement: Supplementary file 2 [file Data_Sheet_2.docx]

Supplementary Materials for

Individual differences in the change of attentional functions with brief one-time focused attention and open monitoring meditations

Masaru Tanaka^1,2,†^, Ryoichi Nakashima^3,4,†^, Kentaro Hiromitsu^1,2,5^, and Hiroshi Imamizu^1,5,6*^

Appendix A. Results of the ANTs

*Table A1.* Mean reaction times (ms) and standard deviations (in parentheses) under each condition across groups for pre- and post-meditation ANT scores (n=32 in each meditation group).

|  |  |  | *FAM* | |  | *OMM* | |  | *noM* | |
| --- | --- | --- | --- | --- | --- | --- | --- | --- | --- | --- |
|  |  |  | Pre | Post |  | Pre | Post |  | Pre | Post |
| No Cue | |  |  |  |  |  |  |  |  |  |
|  | Congruent |  | 527.4 (76.6) | 505.0 (67.6) |  | 535.7 (94.3) | 510.0 (73.7) |  | 525.9 (58.7) | 518.9 (54.6) |
|  | Incongruent | | 619.5 (87.5) | 594.4 (90.7) |  | 614.9 (105.2) | 597.7 (84.6) |  | 604.5 (63.0) | 596.5 (62.9) |
|  | Neutral |  | 519.0 (70.0) | 490.9 (64.8) |  | 522.9 (76.4) | 511.2 (68.4) |  | 515.6 (46.4) | 498.7 (46.8) |
| Centre Cue | |  |  |  |  |  |  |  |  |  |
|  | Congruent |  | 489.4 (73.9) | 456.7 (54.3) |  | 499.4 (89.5) | 471.2 (63.2) |  | 483.4 (47.9) | 462.2 (44.0) |
|  | Incongruent | | 598.2 (84.5) | 566.3 (72.6) |  | 631.5 (194.7) | 567.2 (80.9) |  | 594.6 (59.8) | 579.9 (69.7) |
|  | Neutral |  | 473.8 (59.1) | 452.0 (53.6) |  | 513.1 (193.6) | 470.1 (89.8) |  | 484.9 (48.0) | 463.6 (49.7) |
| Double Cue | |  |  |  |  |  |  |  |  |  |
|  | Congruent |  | 489.0 (63.4) | 458.2 (61.0) |  | 507.6 (107.5) | 472.2 (61.7) |  | 489.6 (54.9) | 471.9 (50.8) |
|  | Incongruent | | 600.2 (93.7) | 559.7 (82.1) |  | 593.7 (110.1) | 566.4 (89.7) |  | 584.2 (56.8) | 574.8 (71.0) |
|  | Neutral |  | 481.6 (58.2) | 455.7 (50.0) |  | 480.5 (78.8) | 467.9 (81.9) |  | 483.3 (46.6) | 461.3 (39.6) |
| Spatial Cue | |  |  |  |  |  |  |  |  |  |
|  | Congruent |  | 439.0 (59.5) | 412.1 (58.7) |  | 459.4 (112.2) | 425.3 (73.5) |  | 433.4 (63.9) | 408.8 (35.2) |
|  | Incongruent | | 530.1 (91.2) | 491.6 (73.9) |  | 536.1 (120.5) | 501.6 (72.9) |  | 515.5 (64.5) | 507.8 (63.9) |
|  | Neutral |  | 432.0 (60.4) | 406.5 (48.8) |  | 442.0 (77.2) | 425.5 (66.3) |  | 425.2 (38.0) | 408.9 (34.4) |

*Table A2.* Mean accuracies (%) and standard deviations (in parentheses) under every condition across groups for pre- and post-meditation ANT scores (n=32 in each meditation group).

|  |  |  | *FAM* | |  | *OMM* | |  | *noM* | |
| --- | --- | --- | --- | --- | --- | --- | --- | --- | --- | --- |
|  |  |  | Pre | Post |  | Pre | Post |  | Pre | Post |
| No Cue | |  |  |  |  |  |  |  |  |  |
|  | Congruent |  | 99.2 (2.0) | 99.1 (1.8) |  | 99.2 (2.0) | 99.5 (1.8) |  | 99.6 (1.6) | 99.9 (0.8) |
|  | Incongruent | | 95.5 (5.5) | 94.3 (6.0) |  | 94.2 (8.6) | 93.1 (6.5) |  | 95.0 (6.9) | 94.0 (8.2) |
|  | Neutral |  | 99.2 (2.3) | 99.5 (1.8) |  | 98.5 (3.2) | 99.6 (1.3) |  | 99.7 (1.0) | 99.3 (1.6) |
| Centre Cue | |  |  |  |  |  |  |  |  |  |
|  | Congruent |  | 99.3 (2.6) | 99.3 (2.2) |  | 99.5 (1.8) | 99.5 (1.4) |  | 99.9 (0.7) | 99.6 (1.3) |
|  | Incongruent | | 91.8 (10.0) | 91.9 (9.3) |  | 91.8 (8.3) | 90.7 (10.0) |  | 91.4 (7.5) | 91.1 (7.8) |
|  | Neutral |  | 99.1 (2.8) | 98.8 (2.5) |  | 99.6 (1.3) | 99.7 (1.0) |  | 99.9 (0.8) | 98.9 (2.4) |
| Double Cue | |  |  |  |  |  |  |  |  |  |
|  | Congruent |  | 99.6 (1.3) | 99.6 (1.3) |  | 99.5 (1.8) | 99.6 (1.3) |  | 99.7 (1.0) | 99.9 (0.8) |
|  | Incongruent | | 94.5 (8.3) | 93.6 (7.0) |  | 90.6 (10.0) | 90.9 (10.0) |  | 99.7 (9.6) | 92.4 (9.7) |
|  | Neutral |  | 99.5 (1.8) | 98.7 (2.7) |  | 99.3 (1.9) | 99.3 (1.6) |  | 99.7 (1.8) | 99.7 (1.0) |
| Spatial Cue | |  |  |  |  |  |  |  |  |  |
|  | Congruent |  | 99.7 (1.0) | 99.6 (1.3) |  | 99.9 (0.7) | 99.9 (0.8) |  | 99.9 (0.7) | 100.0 (0.0) |
|  | Incongruent | | 97.5 (3.8) | 95.8 (5.8) |  | 95.9 (6.1) | 94.7 (5.0) |  | 95.1 (6.2) | 94.5 (6.6) |
|  | Neutral |  | 99.5 (1.8) | 99.3 (1.6) |  | 99.6 (1.3) | 99.3 (1.9) |  | 99.9 (0.7) | 99.9 (0.8) |

Appendix B. ANOVAs for the data of all participants


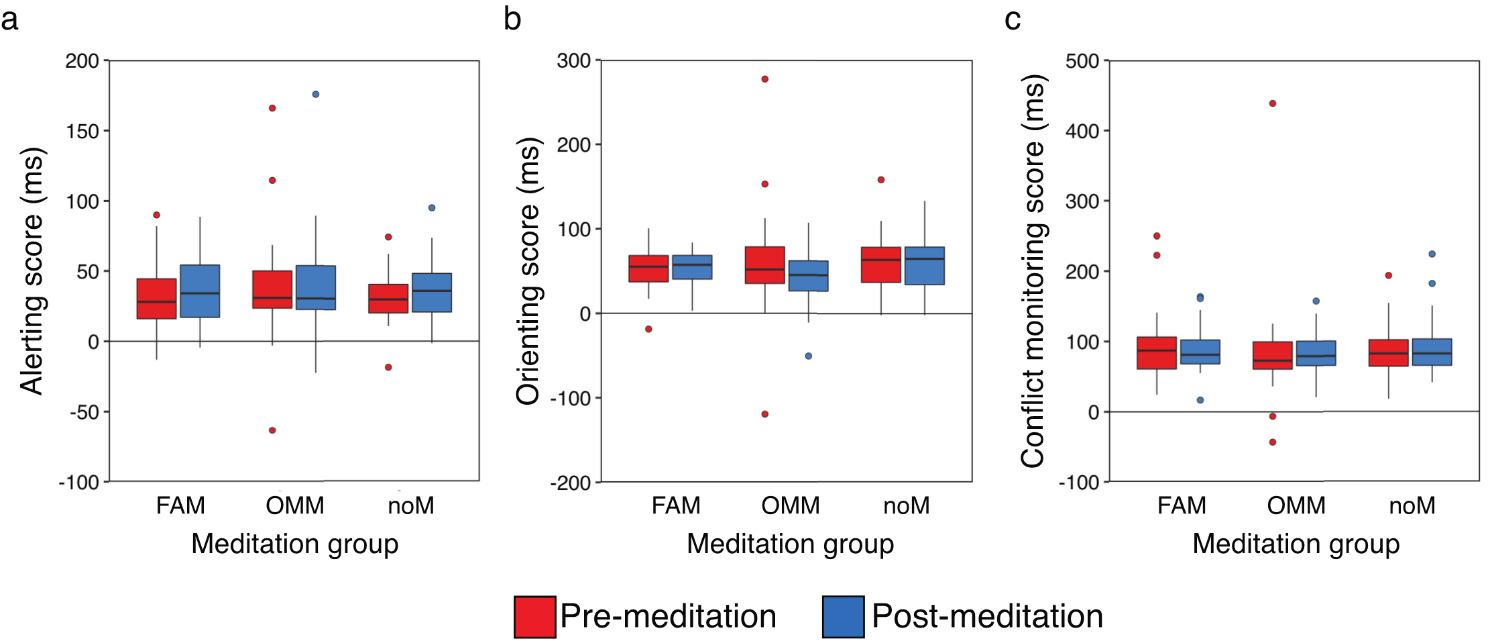


*Fig. S1.* Attentional function scores in pre- and post-meditation ANTs for the three meditation groups (n = 32 in each group), FAM: focused attention meditation, OMM: open-monitoring meditation, noM: no meditation. (**a**) Alerting scores. (**b**) Orienting scores. (**c**) Conflict monitoring scores. Red and blue boxes and circles show the results of the pre- and post-meditation ANT, respectively. In each box-and-whisker plot, the central horizontal line indicates the median, and the bottom and top edges indicate the first and third quartiles, respectively. The upper whisker extends from the edge to the largest value within 1.5 times the interquartile range (IQR) from the edge. The lower whisker extends from the edge to the smallest value within 1.5 times the IQR of the edge. Data points beyond the ends of the whiskers are plotted individually

We conducted ANOVAs using data of all participants (n=32 in each group). For alerting, there were no significant main effects of time, *F*_(1,93)_ = 3.08, *p* = .083, *η_p_^2^* = .033, meditation group, *F*_(2,93)_ = 0.45, *p* = .642, *η_p_^2^* = .010, and no interaction, *F*_(2,93)_ = 0.06, *p* = .938, *η_p_^2^* = .001. For orienting, there were no significant main effects of time, *F*_(1,93)_ = 0.83, *p* = .366, *η_p_^2^* = .009, or meditation group, *F*_(2,93)_ = 0.89, *p* = .416, *η_p_^2^*= .019, and no interaction, *F*_(2,93)_ = 0.97, *p* = .381, *η_p_^2^*= .021. For conflict monitoring, there were no significant main effects of time, *F*_(1,93)_ = 0.01, *p* = 0.922, *η_p_^2^* = .000, or meditation group, *F*_(2,93)_ = 0.24, *p* = .788, *η_p_^2^* = .005, and no interaction, *F*_(2,93)_ = 0.42, *p* = .658, *η_p_^2^* = .009.

Appendix C. Correlation coefficients in FFMQ and ANT scores

*Table A3.* Correlation coefficients between subscales of the FFMQ in meditation groups.

|  |  | *Observing* | *Nonreactivity* | *Nonjudging* | *Describing* |
| --- | --- | --- | --- | --- | --- |
| *FAM* (n=32) | |  |  |  |  |
|  | Nonreactivity | −.16 |  |  |  |
|  | Nonjudging | −.15 | .37 |  |  |
|  | Describing | .48 | .15 | .12 |  |
|  | Awareness | −.04 | .13 | .23 | .27 |
| *OMM* (n=32) | |  |  |  |  |
|  | Nonreactivity | .28 |  |  |  |
|  | Nonjudging | .07 | .29 |  |  |
|  | Describing | .40 | .13 | .09 |  |
|  | Awareness | −.04 | .13 | .09 | .23 |

*Table A4.* Correlation coefficients between changes in ANT indices (post − pre) and subscales of the FFMQ, using data after removing outliers.

|  |  | *Alerting* | *Orienting* | *Conflict* |
| --- | --- | --- | --- | --- |
| *FAM* | | (n =32) | (n =32) | (n =31) |
|  | Observing | .19 | .17 | .00 |
|  | Nonreactivity | −.38 | −.17 | -.02 |
|  | Nonjudging | −.33 | −.13 | .04 |
|  | Describing | −.02 | −.01 | −.15 |
|  | Awareness | −.17 | −.22 | -.07 |
| *OMM* | | (n =30) | (n =31) | (n =31) |
|  | Observing | .13 | .16 | -.03 |
|  | Nonreactivity | .18 | .15 | −.16 |
|  | Nonjudging | .19 | .34 | .07 |
|  | Describing | .40 | -.14 | -.13 |
|  | Awareness | .08 | -.18 | -.09 |

Appendix D. Regression analyses for the data of all participants

*Table A5.* Results of stepwise linear regressions in the combinations in which changes in ANT score could be predicted by FFMQ factors.

| *Independent variable* |  | *r* | *β* | *R^2^* | *adjR^2^* | *F* | *p* |
| --- | --- | --- | --- | --- | --- | --- | --- |
| (A) Alerting in the FAM group (n=32) | | | | | | | |
| Nonreactivity |  | −.38 | −.38 | .15 | .12 | 5.18 | .030 |
| Nonjudging |  | −.33 |  |  |  |  |  |
| Observing |  | .19 |  |  |  |  |  |
| Awareness |  | −.17 |  |  |  |  |  |
| Describing |  | −.02 |  |  |  |  |  |
| (B) Alerting in the OMM group (n=32) | | | | | | | |
| Describing |  | .49 | .49 | .24 | .21 | 9.26 | .005 |
| Awareness |  | .15 |  |  |  |  |  |
| Nonreactivity |  | −.13 |  |  |  |  |  |
| Nonjudging |  | .05 |  |  |  |  |  |
| Observing |  | .02 |  |  |  |  |  |

Note: In the header column, factors in the FFMQ are arranged in order of the absolute correlation coefficient. The right columns show correlation coefficients (*r*) between changes in the ANT score and factors in the FFMQ, standardised regression coefficients (*β*), coefficient of determinations (*R2*), adjusted coefficient of determinations (*adjR2*), *F*-values and *p*-values. (**a**) In the FAM group, the change in alerting was predicted by *nonreactivity*. (**b**) In the OMM group, the change in alerting was predicted by *describing*.


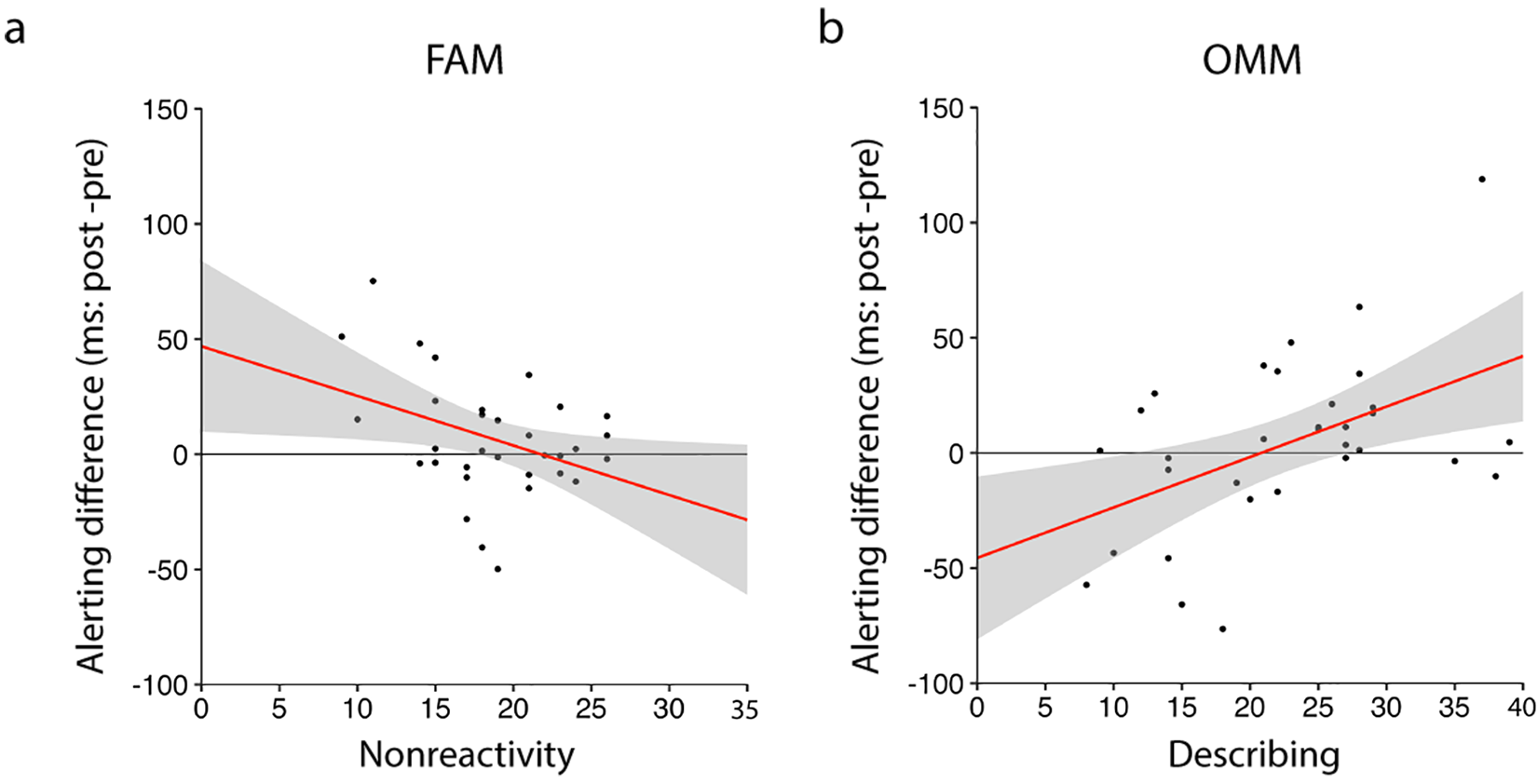


*Fig. S2.* Correlations between changes in ANT score and factors in the FFMQ. (**a**) In the FAM group, the change in the alerting score from pre- to post-meditation was significantly correlated with the nonreactivity score (*r* = −.38, *p* = .030, see also Table A5). (**b**) In the OMM group, the change in the alerting score from pre- to post-meditation was significantly correlated with the describing score (*r* = .49, *p* = .005). Black dots and red lines indicate data of individual participants and linear regressed lines, respectively. Grey shaded areas indicate the 95% confidence intervals of the linear regressed lines.

We conducted the regression analyses for the data of all participants. The results are as follows.

For alerting in the FAM group, only *nonreactivity* was finally adopted as a significant independent variable (Fig. S2a; $alerting difference \left( \mathrm{ms} \right)=-2.15\times nonreactivity+ 46.82$, $R^{2}= .15$, $r=-.38$, $p=.030$). The negative coefficient indicated that the alerting score tended to increase as the *nonreactivity* trait score decreased. For orienting and conflict monitoring in the FAM group, no factor in the FFMQ was adopted as a significant independent variable.

For alerting in the OMM group, only *describing* was finally adopted as a significant independent variable (Fig. S2b; $alerting difference \left( \mathrm{ms} \right)=2.19\times describing-45.47$, $R^{2}=.24$, $r=.49$, $p=.005$). The positive coefficient indicated that the alerting score tended to increase as the *describing* trait score increased. For orienting and conflict monitoring in the OMM group, no factor in the FFMQ was adopted as a significant independent variable.

For orienting and conflict monitoring in the OMM group, no factor in the FFMQ was adopted as a significant independent variable.

*Table A6.* Correlation coefficients between changes in ANT indices (post − pre) and subscales of the FFMQ, using data of all participants.

|  |  | *Alerting* | *Orienting* | *Conflict* |
| --- | --- | --- | --- | --- |
| *FAM* (n=32) | |  |  |  |
|  | Observing | .19 | .17 | −.15 |
|  | Nonreactivity | −.38 | −.17 | .00 |
|  | Nonjudging | −.33 | −.13 | .15 |
|  | Describing | −.02 | −.01 | −.11 |
|  | Awareness | −.17 | −.22 | .09 |
| *OMM* (n=32) | |  |  |  |
|  | Observing | .02 | .31 | .20 |
|  | Nonreactivity | −.13 | .13 | −.05 |
|  | Nonjudging | .05 | .28 | .14 |
|  | Describing | .49 | .15 | .14 |
|  | Awareness | .15 | .05 | .09 |
